# High sperm DNA fragmentation: do we have robust evidence to support antioxidants and testicular sperm extraction to improve fertility outcomes? a narrative review

**DOI:** 10.3389/fendo.2023.1150951

**Published:** 2023-10-05

**Authors:** Massimo Romano, Federico Cirillo, Daria Spadaro, Andrea Busnelli, Stefano Castellano, Elena Albani, Paolo Emanuele Levi-Setti

**Affiliations:** ^1^Department of Gynecology, Division of Gynecology and Reproductive Medicine, Fertility Center, Humanitas Research Hospital, IRCCS, Milan, Italy; ^2^Department of Biomedical Sciences, Humanitas University, Milan, Italy

**Keywords:** sperm DNA fragmentation, sperm dna damage, reactive oxygen species (ROS) levels, antioxidant, Testicular sperm retrieval, sperm DNA in testicular sperm

## Abstract

To date, infertility affects 10% to 15% of couples worldwide. A male factor is estimated to account for up to 50% of cases. Oral supplementation with antioxidants could be helpful to improve sperm quality by reducing oxidative damage. At the same time, there is a growing interest in the literature on the use of testicular sperm in patients with high DNA fragmentation index (DFI). This narrative review aims to evaluate the effectiveness of supplementation of oral antioxidants in infertile men with high DFI compared to testicular sperm retrieval. The current evidence is non-conclusive because of serious risk of bias due to small sample sizes and statistical methods. Further large well-designed randomised placebo-controlled trials are still required to clarify the exact role of these to different therapeutic approaches.

## 1 Introduction

Male infertility accounts for approximately 20-30% of all infertility cases; however, together with female factor infertility it contributes to at least 50% (1). Semen analysis, performed according to World Health Organization (WHO) guidelines, is the gold standard investigation in this field; in particular, in semen analysis the volume, sperm concentration, total sperm count, progressive and total motility and viability are assessed (2).

However, these traditional parameters show relatively poor value in predicting the fertilizing capacity of spermatozoa and reproductive outcomes; indeed, conventional semen analysis does not completely reflect sperm competence (3).

For this reason, in recent years, researches for better diagnostic methods which could predict more precisely male reproductive outcomes resulted in a focus on sperm DNA fragmentation (SDF) (4). Over the past two decades, it has been suggested that the assessment of sperm nuclear DNA integrity is a more objective parameter than conventional semen analysis in evaluating sperm quality. SDF can be defined as any chemical change in the normal structure of the DNA that can affect the genetic material in the form of single or double strand breaks (2). High sperm DNA damage has been reported to associate with reduced fertilization potential, abnormal embryonic development and a worse pregnancy outcome. However, since the degrees of sperm DNA damage was significantly different between fertile and infertile men, it seems reasonable that the utilization of sperm DNA damage can be used as a new potential fertility predictor (5).

Morphological defects have been associated with elevated DNA fragmentation or with alteration of chromosomal structure, aneuploidy and incomplete chromatin maturation and aneuploidy, but not always basal features of the sperm valuation can predict an alteration of double strand DNA (2).

There is evidence supporting the presence of a higher degree of sperm DNA damage in infertile men than in fertile ones (6). This is particularly relevant in an era where Assisted Reproductive Technologies (ART) are commonly employed and where the selection of higher quality spermatozoa could be performed (7). Indeed the 6^th^ WHO laboratory manual for the examination and processing of human semen manual in 2021 dedicates a broader section to the topic of DNA fragmentation than the previous versions. However, despite a large number of studies, there is no consensus on whether or not measuring sperm DNA fragmentation is a predictive and/or prognostic factor for a man’s fertility potential. Furthermore, interlaboratory variation and lack of standardization limit the clinical usefulness of sperm DNA fragmentation (5).

Aetiology of sperm DNA damage is multi-factorial and not completely clear. Oxidative stress and reactive oxygen species (ROS) are considered well-known damaging factors to sperm and responsible for the disruption of the prooxidant-antioxidant balance (8).

An imbalance between ROS production and antioxidant capacity results in increased sperm exposure to ROS, which through multiple pathways - including sperm lipid peroxidation, abortive apoptosis and DNA damage – plays a critical role in altering sperm function (9). Elevated levels of ROS can also affect the passage of sperm via seminiferous tubules and epididymis (10).

The generation of seminal ROS could be attributed to genital tract infection or inflammation, varicocele, testicular torsion and cryptorchidism. Other factors include male aging, exposure to toxic or antiblastic substances, smoking, alcohol abuse (10, 11). Fort these reason, different strategies have been proposed to decrease ROS production and increase antioxidant capacity: these approaches go from lifestyle changes to antioxidant therapies (12). For this reason, antioxidants represent an adjuvant treatment in such cases, avoiding further damage caused by ROS and independently from their antioxidant properties, the activities of these molecules have been widely investigated and described.

On the other hand, in infertile men undertaking ART cycles, a strategy proposed in order to overcome DNA damage associated with the passage of sperm through the seminiferous tubules and epididymis is the use of testicular sperm extraction (TESE) (13). The significantly lower incidence of DNA damage in testicular retrieved spermatozoa with respect to ejaculated ones, supported the clinical role of this procedure in patients diagnosed with high SDF (14).

Although the fertilization ability of sperm with elevated SDF may not be impaired, multiple recent meta-analyses suggested its role in affecting embryo development, implantation, and pregnancies in both natural and assisted reproduction (15). Moreover, even if SDF is prevalent among men with abnormal ejaculate parameters, it has been proposed to be related to infertility also in normozoospermic individuals, becoming an important additional test in male infertility work-up and a promising biomarker in basic and clinical andrology (16).

For this reason, the focus of this review is to examine the different therapeutical strategies to overcome sperm DNA damage in infertile men undertaking ART cycles.

## 2 Materials and methods

The present review is based on a bibliographic search performed in November 2022, in PubMed, Google Scholar and Cochrane Library, of studies published in English using the following terms or text strings: sperm DNA fragmentation, sperm DNA integrity, sperm DNA damage, SDF, DNA Fragmentation Index (DFI), reactive oxygen species (ROS) levels, antioxidant, TESE, testicular sperm retrieval, sperm DNA in testicular sperm.

## 3 Findings

### 3.1 Antioxidant therapy

Antioxidant therapy could be useful in reducing general oxidative stress and has been considered for oral supplementation and introduced into clinical practice for the treatment of male infertility (13).

The main antioxidants include Coenzyme Q10, L-Carnitine, Zinc, Vitamin D, Vitamin E, N -Acetyl-Cysteine. Principal studies are summarized in **Table 1**.

a) The potential role of Coenzyme Q10 (CoQ10) on male fertility and sperm DNA fragmentation was widely investigated (39). CoQ10 is a constituent of mitochondrial membrane, involved in the lipid and DNA oxidation. It has a specific role in inhibition of superoxide production, even in the sperm membrane (17, 18, 39).

**Table 1:** Antioxidants and their mechanism of action.

Molecule	Definition	Action	Effect on sperm	Supporting Studies
Coenzyme Q10	Constituent of mitochondrial membrane	Inhibition of superoxide production	DNA fragmentation reduction and increase in sperm cells numeber and quality	(17–21)
L-carnitine	Hydrophilic amino acid derived from amino acid lysin	Promotion of the entry of long-chain fatty acids into mitochondria	Improvement of sperm volume, progressive motility and vitality; reduction of SDF	(21–23)
Zinc	Divalent metal	Catalytic action of copper/zinc-superoxide dismutase, stabilization of membrane structure	Improvement of sperm paramenters and reduction in the incidence of antisperm antibodies	(24–28)
Vitamin D	Fat-soluble vitamin	Inhibition of lipid peroxidation	Increase in sperm progressive motility and reduction in SDF	(29–33)
Vitamin E	Fat-soluble vitamin	Reduction of hydroxyl radicals and lipid peroxidation	Improvement in sperm motility and reduction in SDF	(26, 34, 35)
N-acetyl-L-cysteine	Modified amino acid derived from L-cysteine	Scavenger of free radicals, improves glutathione (GSH) levels	Improvement of sperm parameters, reduction in SDF	(36–38)

Several studies have reported that low level of CoQ10 could be associated with worse sperm quality and high sperm DNA damage. In 2015 some authors treated patients with low-grade varicocele (I grade) with multivitamins and CoQ10. After treatment, patients showed an average relative reduction of 22.1% in sperm DNA fragmentation and an improvement in terms of total number of sperm cells (19).

Similarly, Nadjarzadeh et al. found out a significant positive correlation between CoQ10 concentration and normal sperm morphology, catalase and SOD (P < 0.001), concluding that three-month supplementation with CoQ10 in infertile men can attenuate oxidative stress in seminal plasma and improve semen parameters and antioxidant enzymes’ activity (20).

A recent prospective study involving 93 infertile males with a history of failed IVF/ICSI compared with 10 healthy male volunteers as controls concluded that a 3-month lifestyle’s intervention program added to antioxidant therapy reduces oxidative stress and DFI (21).

These finding are in line with the results of a recent randomized controlled trial on 65 infertile men treated with CoQ10 therapy for 3 months: this study highlighted a statistically significant improvement in seminal basal parameters and DFI compared with 40 fertile men (40). In a recent review Lucignani et al. concluded that the effect of CoQ10 and its improving role on semen parameters and sperm DFI, even in single administration or in couple with melatonin, resulting in an ameliorated FR in in IVF/ICSI (41).

Further in-depth interventions are needed to reveal the exact mechanism of action of CoQ10 and to determine the appropriate standardized dose and duration of CoQ10 supplementation in the treatment of specific male infertility cases. Additional evidence is needed to further support the treatment of male infertility with melatonin supplementation.

This improvement was also demonstrated in *in-vitro* incubation of semen with CoQ10: sperm incubated with CoQ10 resulted in a significant increase in the sperm parameters. A significant difference in the percentage of the DNA fragmentation, sperm apoptosis, oxidative sperm test and sperm mitochondrial activity registered (22).

Therefore, supplementation with CoQ10 decreases SDF, improves semen quality and seminal antioxidant capacity. However, more well-performed and wide RCT on the positive role of CoQ10 are needed to clarify the potential improvement of this approach, especially in oligo-asthenozoospermic patients.

b) L-carnitine (LC) is a hydrophilic amino acid derived from the amino acid lysin and its main role is to facilitate and increase the entry of long-chain fatty acids into the mitochondria. It is produced in the epididymis and it is transported to spermatozoa, where it increases sperm motility (23, 24). Furthermore, LC has also antioxidant activity both *in vitro* and *in vivo* reducing oxidative stress that causes DNA damage (24).

As Coenzyme Q10, L-Carnitine incubation allows to improve sperm quality during, before and after cryopreservation in oligospermic patients (22). It’s crucial to know that during cryopreservation, the oxidative stress mediates mitochondrial dysfunction. In frozen-thawed semen, staining with C11-BODIPY581/591 revealed that the sperm mid-piece is most affected by oxidative stress, followed by the tail plasma membrane and least peroxidation in the head region (25). The mitochondrial dysfunction directly alters the inner or outer mitochondrial membrane, damages mitochondrial DNA or results in the loss of the dense sheath around the mitochondria. However, it indirectly exaggerates nuclear DNA fragmentation and affects production of specific mitochondrial proteins which are coded by nuclear genome (26).

Regarding oral supplementation, in a prospective, randomised, double-blind, placebo-controlled clinical trial, Micic et al. enrolled 175 males (19-44 years) with idiopathic oligoasthenozoospermia and primary infertility (27). These patients received a combination of L‐carnitine and L‐acetylcarnitine with micronutrients vs. placebo for 3 and 6 months. The authors pointed out a statistically significant improvement in terms of sperm volume, progressive motility and vitality after 6 months compared to the beginning. Moreover, the sperm DNA fragmentation index significantly decreased compared to baseline.

These data were strongly in line with another recent open, prospective, randomized trial involving 114 men after microsurgical varicocelectomy (MVE) (28). These patients received a complex of acetyl-L-carnitine, L-carnitine fumarate and alpha-lipoic acid after surgery vs. placebo. The efficacy was proved after 3 months by testing standard semen parameters and the level of sperm DNA fragmentation. To measure oxidative stress the authors used the level of free oxygen radicals, highlighting how it significantly decreased (86%) in the treated group compared to the placebo. Therefore, a deeper decrease in DNA fragmentation was seen in the treated patients compared to the placebos (21.5% vs. 3.6%).

c) The positive effects of **zinc** on semen parameters have been known for some time and have been documented in clinical studies. Zinc has a membrane-stabilizing and antioxidant activity and maintains sperm viability by inhibiting DNAases (42).

In 1998, Omu et al. randomly divided 100 men with asthenozoospermia into two groups: 250 mg twice daily zinc therapy vs. placebo (43). Treatment lasted 3 months and patients were tested after 6 months. Sperm parameters, antisperm antibodies (ASAs), sex hormones and cytokines (interleukin-4 – IL-4) were evaluated before and after treatment. The authors registered a significant improvement in sperm quality in terms of sperm count and progressive motility and a reduction in the incidence of ASAs. IL-4 was statistically higher after zinc therapy (P<.02), proving the anti-inflammatory effect of this therapy (29).

In 2008 the same authors randomized forty-five men with asthenozoospermia into four therapy groups: zinc only or in combination with vitamin A or E vs. non-therapy control group. They demonstrated that zinc therapy, alone or in combination with vitamins, was associated with improved sperm parameters and with reduced oxidative stress and sperm DNA fragmentation index (DFI) (30).

Moreover, in 2017 Isaac and colleagues reported that supplementing zinc oxide nanoparticles in sperm cryopreservation medium minimizes the freeze-thaw-induced damage to spermatozoa (31).

In 2013, Raigani investigated the improvement of sperm function in 83 subfertile oligoasthenoteratozoospermic (OAT) men in a 16-week intervention randomised, double-blind clinical trial with daily treatment of folic acid (5 mg day) and zinc sulphate (220 mg day), versus placebo. In the zinc sulphate/placebo group, sperm chromatin integrity analysis showed a significant reduction in chromatin abnormality (45.5 ± 13.2 vs 40.2 ± 18.3) (P = 0.048) (32).

In 2022, Dadgar et al. randomized men with idiopathic infertility, dividing them into four groups: pentoxifylline, zinc, pentoxifylline + zinc, and placebo. In the zinc and zinc + pentoxifylline groups, an improvement in terms of morphology and sperm DNA fragmentation was observed after 3 months of therapy (44).

d) Vitamin supplementation is also considered a valid therapy for reducing oxidative stress in patients with high DFI, in particular vitamins D, E and C.

Vitamin D is considered an important micronutrient with many biological effects and its deficiency has become an emerging epidemic over the last decade (33). According to evidence, vitamin D levels seem to be greater in fertile men compared to infertile ones (45). Some authors have hypothesized that vitamin D could have a relevant role in spermatogenesis, acquisition of sperm motility, and endocrine functions improving male fertility overall (46, 47).

Taheri Moghadam et al. (48) evaluated the influence of vitamin D on the survival and integrity of fertile sperm after cryopreservation: motile and viable sperm concentration was substantially higher in treated groups; however, morphological analysis did not show any remarkable change. Similarly, vitamin D strongly reduced lipid peroxidation values through Tunel test and markers of apoptosis were considerably lower in treated groups (P-value<0.05).

Deng XL et al. showed that 3 months integration with both Vitamin D and calcium in infertile patients with OAT strongly increased sperm progressive motility and pregnancy rate in the treated group (34). Moreover, vitamin D deficiency was linked to an increase in sperm DNA fragmentation even in animal studies (35).

Despite this positive theoretical association, there is not a general consensus on the improvement in semen parameters of treated patients. In a randomized, controlled trial, Banks et al. (49) reported that semen parameters and sperm DNA fragmentation did not statistically differ between men with vitamin D deficiency and men with normal vitamin D values. In addition, the clinical pregnancy rates and live birth rates were comparable between therapy and placebo. Likewise, in a cross-sectional study, Rubal confirmed these results, evaluating sperm parameters and DFI in patients with vitamin D deficiency (defined as serum level <20 ng/mL), insufficiency (as 20-30 ng/mL), and abundance (as >30 ng/mL). Comparing the three groups, there was no significant difference in sperm concentration, motility and overall morphology. There was no significant difference in DFI among the vitamin D deficient (17.6%), insufficient (17.4%), and replete (19.5%) groups (50). Likewise, in a case control study, Güngör included 58 infertile men with unexplained infertility and 50 matched fertile men. As expected, DNA damage was found to be significantly higher in the unexplained infertile group (p<0.002), while there was a negative and significant correlation between vitamin D levels and sperm DNA damage (p<0.001). In the logistic regression analysis, serum vitamin D > 20 ng/mL led to an improvement in fertility outcome. In the fertile group, there was no significant correlation between vitamin D levels and sperm DNA damage and other sperm parameters (51).

Also, the effects of vitamin E and its use as an antioxidant are among the topics of interest in the treatment of male infertility. Vitamin E is an organic fat-soluble substance; it is present above all in cell membranes (33). It has the ability to reduce hydroxyl radicals, lowering lipid peroxidation caused by ROS to plasma membranes (36). Some studies have reported a prominent relationship between the levels of vitamin E in seminal plasma and the sperm motility. Furthermore, it was observed that it is more common for infertile patients to have lower levels of vitamin E compared to fertile men (37). Indeed, Comhaire et al. (38) reported a significant reduction in seminal ROS levels in patients treated with a combined therapy of 180 mg vitamin E and 30 mg b-carotene in idiopathic infertile men.

Regarding vitamin C (ascorbic acid), its concentration is almost 10 times higher in the semen plasma than in the blood serum and it neutralizes hydroxyl, superoxide, and peroxide radicals (52). It has been shown that the addition of vitamin C (and vitamin E) to the semen of men with normozoospermia and asthenozoospermia reduces the degree of SDF caused by ROS (37). Greco et al. (53) assessed the utility of 1 g vitamin C plus 1 g vitamin E daily for 2 months vs. placebo in 64 patients with unexplained infertility and elevated levels of DFI. The authors identified significant reduction in the percentage of DFI in the treatment group.

Moreover, Akmal et al. achieved an improvement in total sperm count, motility, and morphology after the administration of vitamin C in the dose of 2000 mg/day (54).

We previously cited the study in which Omu et al. (30) assessed the advantage of daily vitamin E (20 mg), vitamin C (10 mg) and zinc (400 mg) for 3 months on 45 men with low motility. As already reported, the authors confirmed the positive association between this multiple therapy and the reduction of oxidative stress on spermatogenesis, in terms of DFI and basal sperm features. The authors focused the research also in the microscopic modification of the sperm DNA integrity, demonstrating that zinc deficiency was significantly associated (p<0.005) with abnormality of the tail and defect of the flagella and defect of microtubular couples in about 32–34% of the patients with zinc deficiency compared to 12–16% in the treated sample and to control groups.

e) N-acetyl-L-cysteine (NAC) is derived from the amino acid L-cysteine, which has a scavenger activity (55). Thus, it is able to improve glutathione (GSH) levels during oxidative stress (56). NAC is one of the most powerful and commonly used antioxidants, as it is often prescribed in the treatment of various diseases, such as respiratory infections, heart diseases, and epilepsy (57). These antioxidant properties of NAC were also reported in the context of its influence on germ cell survival. *In-vitro* studies have also demonstrated a significant decrement in ROS levels and an improvement in sperm motility after sample incubation with NAC (58).

In a prospective clinical trial, Barekat et al. (59) included 35 infertile men with varicocele randomly divided into control (n=20) and NAC (n=15) groups. The authors assessed semen parameters and oxidative stress before and three months after varicocelectomy. A significant decrease in the percentage of abnormal semen parameters, DNA fragmentation and oxidative stress were assessed in treated group compared to control.

In 2019 Jannatifar (58) investigated the role of supplementation with NAC on the sperm quality, integrity of DNA chromatin, and levels of ROS in infertile patients. The authors administered oral therapy composed by 600mg/day of NAC to 50 infertile men with asthenoteratozoospermia. The treatment lasted for 3 months and the results were compared with pre-treatment status. After treatment, patients’ sperm count and motility increased significantly and, at the same time, abnormal morphology and DNA fragmentation index showed significant decreases compared to pre-treatment levels (p < 0.05).

Furthermore, a double-blind, placebo-controlled trial randomized 468 infertile men with idiopathic oligo-asthenoteratospermia (60). The study’s populationwas divided into 4 grous: 200 microg selenium/day (116 patients), 600 mg N-acetyl-cysteine/day (118 patients), both (selenium plus N-acetyl-cysteine - 116 patients) placebo (control group of 118 patients). The treatments lasted 26 weeks and the semen tests were repeated after a 30-week treatment-free period. The authors documented a significant and statistically strong correlation between the blood level of selenium and N-acetyl-cysteine and the features of sperm quality in terms of sperm concentration (r = 0.67, p = 0.01), motility (r = 0.64, p = 0.01), and normal morphology (r = 0.66, p = 0.01).

Furthermore, sperm incubation with NAC seems to play a defensive role in a dose-dependent way in the alterations of seminiferous tubule of the spermatozoa and also inhibiting the death of the sperm precursor, as demonstrated by Erkkila et al. in 1998 using light and electronic microscopy.

We summarized the principle cited studies in **Table 2**.

**Table 2:** Principal studies for antioxidant(s) treatment, for Sperm DNA fragmentation.

Study	Participant	RCT	Interventions	Treatment period	Outcomes
Josep Gual-Frau (2015) (19)	Infertile grade I varicocele patients	no	Multavitamins with CoQ10	3 mo	Total numbers of sperm cells were increased. Sperm DNA integrity in grade I varicocele patients may be improved by oral antioxidant treatment.
Nadjarzadeh (2013) (20)	Infertile men with idiopathic oligoasthenoteratozoospermia	yes	200 mg daily of CoQ10 vs placebo	3 mo	Supplementation with CoQ10 in OAT infertile men can attenuate oxidative stress in seminal plasma and improve semen parameters and antioxidant enzymes activity.
Humaidan (2022) (21)	Infertile males with a history of failed IVF/ICSI.	no	Diet and exercise, combined with oral antioxidant (multivitamins, coQ10, omega-3, and oligo-elements).	3 mo	A personalized lifestyle and antioxidant intervention could improve fertility of subfertile couples through a reduction in DFI.
Alahmar (2021) (40)	infertile patients with idiopathic OA vs fertile men	yes	CoQ10 at the dose of 200 mg/d orally	3 mo	CoQ10 supplementation for three months could improve semen parameters, oxidative stress markers and reduce SDF in infertile men with idiopathic OA
Nezhad (2021) (22)	Semen was collected from oligospermic men	no	The samples of each individual were divided and incubated with different anti-oxidant vs placebo	/	Antioxidant treatments significantly reduced the number of ROS + in the pre and post freezing groups. Antioxidants also reduced the percentage of DNA fragmentation and protamine deficiency in pre-and post-freezing.
Micic (2019) (27).	infertile men with idiopathic oligoasthenoteratospermia	Yes	L‐carnitine and L‐acetylcarnitine with micronutrients	3 mo vs 6 mo vs placebo	Sperm volume, progressive motility and vitality and sperm DFI significantly improved after 6 months compared to baseline.
Gamidov (2019) (28)	infertile men with oligoasthenoteratospermia, underwent to microsurgical varicocelectomy	Yes	Complex of acetyl-L-carnitine, L-carnitine fumarate and alpha-lipoic acid after surgery vs placebo	3 mo	Improvement of basic sperm parameters (sperm concentration and motility) and a decrease in the level of sperm DNA fragmentation in the short term.
Omu 1998 (43)	men with idiopathic asthenozoospermia	yes	250 mg twice daily zinc therapy for 3 months vs no therapy	3 mo	Sperm parameters, circulating antisperm antibodies, sex hormones and T helper cytokines were evaluated before and after treatment for the two groups
Omu (2008) (30)	forty-five men with asthenozoospermia	yes	four therapy groups: zinc only: n = 11; zinc + vitamin E: n = 12 and zinc + vitamins E + C: n = 14 for 3 months, and non-therapy control group: n = 8	3 mo	zinc therapy alone or in combination with vitamin were associated with improved sperm parameters with less oxidative stress and sperm DNA fragmentation index (DFI).
Raigani (2013) (32)	Subfertile oligoasthenoteratozoospermic (OAT) men	yes	daily treatment of folic acid (5 mg day) and zinc sulphate (220 mg day),	16 weeks	Sperm chromatin integrity increased significantly in subfertile men receiving only zinc sulphate treatment (P = 0.048)
Dadgar (2022) (44)	men with idiopathic infertility	yes	four groups: pentoxifylline, zinc, pentoxifylline + zinc, and placebo	3 mo	A significant improvement in term of morphology and sperm DNA fragmentation was observed.
Banks (2021) (49)	Men with idiopathic OAT	yes	vitamin formulation including vitamin D 2,000 IU daily or placebo	6 mo	Semen parameters and DFI were not statistically significantly different between men with vitamin D deficiency and men with 25(OH)D levels ≥20 ng/mL. In addition, clinical pregnancy and live birth rates were similar.
Gungor	men with unexplained infertility	no	correlation between vit D levels and sperm DNA damage	/	There was a negative and significant correlation between vit D levels and sperm DNA damage (p<0.001). serum vit D > 20 ng/mL led to an improvement in fertility outcome
Taheri Moghadam (2019) (48)	Fertile men	no	integrity of fertile sperm after cryopreservation with vitamin D supplementation	/	ROS and lipid peroxidation values were dramatically reduced by vitamin D as well as TUNEL and Annexin assay for apoptosis were considerably lower in treated groups (P-value<0.05).
Deng (2014) (33)	men with idiopathic oligoasthenozoospermi	yes	oral VD 200 IU/d and calcium 600 mg/d; while the latter administered oral vitamin E 100 mg and vitamin C 100 mg	3 mo	Vitamin D and calcium in oligo-asthenozoospermia infertile men significantly increased sperm progressive motility and rate of pregnancy in the intervention group
Comhaire (2000) (38)	Idiopathic infertile men	no	oral antioxidants (N-acetyl-cysteine or vitamins A plus E) and essential fatty acids (FA)	6 mo	The treatment significantly reduced ROS, increased the Acrosome Reaction, the proportion of polyunsaturated FA of the phospholipids, and sperm membrane fluidity.
Greco (2005) (53)	men with unexplained infertility and an elevated (> or = 15%) percentage of DNA-fragmented spermatozoa in the ejaculate	yes	Daily administration of 1 g vitamin C+ 1 g vitamin E vs placebo	2 mo	the percentage of DNA-fragmented spermatozoa was markedly reduced (P <.001) in the antioxidant treatment group after the treatment (9.1 +/- 7.2) as compared with the pretreatment values (22.1 +/- 7.7)
Barekat (2016) (59)	Prospective clinical trial included infertile men with varicocelectomy randomly divided into control and NAC groups	yes	three tablets of NAC (200 mg daily)	3 mo	Percentage of protamine deficiency and DNA fragmentation significantly differed between the NAC and control groups
Jannatifar (2019) (58)	infertile men with asthenoteratozoospermia	yes	NAC (600 mg/d)	3 mo	Patients’ sperm count and motility increased significantly; DNA fragmentation and protamine deficiency showed significant decreases compared to pre-treatment levels (P < 0.05). Hormonal profile improvement was associated with lowered FSH and LH levels and increased amount of testosterone (P < 0.05)
Safarinejad (2009) (60)	infertile men with idiopathic oligo-asthenoteratospermia	yes	200 microg selenium orally daily (selenium group of 116), 600 mg N-acetyl-cysteine orally daily (N-acetyl-cysteine group of 118), 200 microg selenium plus 600 mg N-acetyl-cysteine orally daily (selenium plus N-acetyl-cysteine group of 116)	26 weeks	The authors observed a significant positive correlation between the selenium and N-acetyl-cysteine concentrations, and mean sperm concentration (r = 0.67, p = 0.01), sperm motility (r = 0.64, p = 0.01) and percent normal morphology (r = 0.66, p = 0.01).

### 3.2 Testicular sperm retrieval and sperm DNA damage

In assisted reproductive techniques (ART), damage in spermatozoa DNA was ascribed as being responsible for reduced rates of fertilization, impaired embryo development and higher rates of early pregnancy loss (9, 61).

Therefore, in infertile men undertaking ART cycles, a strategy proposed in order to bypass DNA damage that occurred during the passage through the seminiferous tubules and epididymis was the use of testicular sperm.

By comparing the incidence of DNA fragmentation in ejaculated versus testicular sperm, this incidence was found to be significantly lower in the testicular samples (4.8 ± 3. % versus 23.6 ± 5.1% (14) and 39.7% ± 14.8 versus 13.3% ± 7.3 (62).

A prospective study enrolling a total of 147 couples undergoing IVF-ICSI cycles and in which the male partner was diagnosed with oligospermia and high SDF, a significantly lower DNA fragmentation index (DFI) was detected in testicular sperm with respect to ejaculated one (8.3% in testicular sperm versus 40.7% in ejaculated sperm). Moreover, also a significant improvement in the percentage of live birth and of miscarriage following the procedure was observed, with a relative risk for miscarriage of 0.29 and of 1.76 for live birth (63).

The degree of sperm DNA fragmentation was directly linked with pregnancy outcomes: in couples who failed previous cycles of IVF-ICSI and where the male partner was diagnosed with elevated TUNEL-positive ejaculated sperm, the employment of testicular sperm for ICSI increased the rate of live birth and pregnancy by 50% (64).

In addition, using testicular sperm instead of ejaculated one for ICSI improves pregnancy outcomes also in patients with normal range conventional sperm parameters and high DFI, with an observed clinical pregnancy of 13% vs 8% (p value 0.045) and an ongoing pregnancy of 12% vs 6% (p value 0.023) (65).

In this respect, also superior quality evidence studies documented a higher clinical pregnancy and live birth rate in ICSI cycles in which testicular sperm of men with confirmed post-testicular SDF was used (66). However, patients of the two compared groups (testicular versus ejaculated ICSI) were not randomized, determining a selection bias. Moreover, a major limitation of this study is the exclusion of relevant factors - as maternal age, infertility duration or number of oocytes retrieved – which might influence the ICSI outcome.

The significant association between the degree of SDF and adverse pregnancy outcomes was suggested by a recent meta-analysis, which demonstrated that couples with unexplained recurrent miscarriage showed higher degree of SDF than couples without this condition (67).

In the light of this data, some authors proposed to use TESE to reduce SDF, improving the obstetric outcomes. TESE was demonstrated to be superior to other interventions investigated - as PICSI or IMSI - in improving pregnancy outcomes in patients with high seminal SDF (68). However, it is of relevance to note that if on one side the ICSI outcomes amelioration linked to the employment of testicular sperm positively affected clinical pregnancy and implantation rates, fertilization rate did not change significantly (14, 69).

In contrast to the studies presented above which are in favor of a positive correlation between TESE and an improvement in clinical pregnancy rate, live birth rate and miscarriage rate, conflicting data which did not demonstrate any significant difference in these pregnancy outcomes between ejaculated versus testicular ICSI exist (70).

Notwithstanding the lower degree of DNA damage, sperm retrieval from testis may be associated with a higher rate of aneuploidy: in fact, comparing the ejaculated and the testicular spermatozoa obtained from the same patients, a twofold increase in total aneuploidy was demonstrated in testicular spermatozoa with respect to ejaculated ones, with a higher rate of aneuploidy for chromosomes 18, 21, X and Y (71); however, current evidence did not demonstrate a greater risk of malformations in embryos obtained through this technique (72). Furthermore, despite the improvements brought using testicular sperm for ICSI, surgical sperm retrieval represents an invasive procedure and, therefore, it is associated with potential complications as persistent pain, swelling, infection and hydrocele (73). In patients who underwent TESE, ultrasounds performed 3 months after the procedure showed intratesticular hematomas in 80% of cases (74).

In addition, testicular devascularization and tissue over-removal in large-volume conventional TESE can determine a decrease in serum testosterone, which may eventually become permanent (75, 76).

In conclusion, in the light of the available data, testicular ICSI should be preferred in men with significant SDF with history of recurrent ICSI failures and when other measures to correct SDF causes have failed (66).

We have summarized the main cited studies in **Table 3** below.

**Table 3:** Testicular sperm retrieval for high sperm DNA fragmentation.

Study	Participants	Design	SDF testing method	Interventions	Outcome
Greco et al., 2005 (14)	18 couples ≥ 2 ICSI failure with ejaculated sperm	Case-control study	TUNEL assay	TESE and TESA	SDF rate in testicular vs ejaculated sperm. Fertilization rate, cleavage rate, implantation rate, clinical pregnancy rate
Moskovtsev et al., 2010 (62)	12 infertile men with high DNA damage despite antioxidant therapy	Prospective cohort study	TUNEL assay	TESE	SDF rate in testicular vs ejaculated sperm
Moskovtsev et al., 2012 (71)	8 infertile men with high DNA damage despite antioxidant therapy	Prospective cohort study	TUNEL assay		SDF rate in testicular vs ejaculated sperm and sperm aneuploidy rate
Mehta et al., 2015 (64)	24 oligospermic men who failed ≥ ART cycles using ejaculated sperm with TUNEL-positive proportion > 7%	Retrospective cohort	TUNEL assay	Micro-TESE	SDF rate in testicular vs ejaculated sperm CPR in the testicular sperm group
Esteves et al., 2015 (63)	147 oligospermic men with > 30% SDF	Prospective cohort study	SCD	TESE and TESA	SDF rate in testicular vs ejaculated sperm. Clinical pregnancy, miscarriage rate and live birth rate
Pabuccu et al., 2016 (65)	71 normozoospermic men with DFI > 30% and who failed ≥ 2 ART cycles	Retrospective cohort	SCD	TESA	Fertilization rate, implantation rate, CPR, OPR
Bradley et al., 2016 (68)	1924 men with SDF > 30%	Retrospective cohort	SCIT		Fertilization rate, CPR, implantation rate, miscarriage rate, LBR
Arafa et al., 2017 (69)	36 men with high SDF with a previous failed ejaculated sperm-ICSI cycle	Prospective cohort study	SCD	TESA	Fertilization rate, pregnancy loss, Clinical pregnancy rate and live birth rate
Alharbi et al., 2020 (70)	52 nonazospermic men with SDF > 15% and >30% that had T-ICSI after ≥ 1 Ej-ICSI	Retrospective cohort	SCSA	TESA	CPR, miscarriage rate and LBR

## 4 Discussion

In the last decades significant progress has been made in reproductive medicine. Unfortunately, numerous basic, clinical, and scientific questions in male fertility have still remained unanswered (77).

This is symptomatic of an ‘andrological ignorance’: in contrast with the female counterpart, for whom there are multiple effective options for both infertility and contraception, the treatments for males are lacking (78).

In fact, in the early 1950s, a series of investigations on the quality of human semen was published, paving the way for the first hints of modern andrology (2).

From 1992, after the birth of the first ICSI-conceived child, the ‘use and abuse’ of this technique has significantly increased, and treatments are used even when no male problem is present. So, ICSI treatment ironically took over: its success has moved research attention on the female factor, shifting the focus from the diagnosis and treatment of male infertility.

Nonetheless, the spermatozoa heavily participate in the genetic, epigenetic, and cellular generation of the embryo. On the basis of this assumption, sperm expression in ART techniques has led to a growing interest, as it has been additionally suggested by the identification of the fertilization rate as a key indicator of the IVF laboratory’s performance and as a novel indicator for cumulative live birth rate (79).

Given these considerations, since 1980, the WHO has published other five editions of the manual containing standardized methods for testing semen (WHO 1987, 1992, 1999, 2010, 2021), bringing the matter as close as possible to other already standardized laboratory diagnostic practices (80–84).

As already described, one of the most recent tests introduced on male fertility and sperm fertilization ability is the sperm DNA fragmentation test. Sperm DNA damage can be defined as any chemical change in the normal structure of the DNA (2). Among these changes of the genetic material, sperm DNA fragmentation (sDF) is one of the most common disturbances. In the early 1980s, the correlation between sperm DNA fragmentation and infertility was introduced, and SCSA(sperm chromatin structure analysis) was performed as the first DNA fragmentation test (85).

To date, several tests have been developed to evaluate sperm DNA fragmentation: Sperm Chromatin Dispersion test (SCD), TUNEL (TdT-mediated dUTP nick-end labeling), and Comet Assay. TUNEL and Comet Assay directly assess DNA damage, while SCSA and SCD measure DNA fragmentation after a denaturation process; furthermore, the use of flow cytometry in TUNEL and SCSA allows a low intra-operator variability, differently from the SCD test which can be affected by the laboratorist’s experience. Unfortunately, all these tests remain complex, expensive, and not equally distributed in every laboratory (2).

The principal mechanisms most clearly associated with DNA damage are oxidative stress, defective chromatin formation of seminal DNA, and apoptosis, but very often it is difficult to define the ‘primum movens’.

Reactive oxygen species (ROS) are produced during normal cellular metabolism and they are essential for normal sperm activity (like capacitation, acrosome reaction, and fertilization) (86). Oxidative stress happens when the production of ROS overlaps the natural antioxidant capacity, leading to cellular insults: excess of ROS affects the cell membrane, invalidating sperm motility and the ability to fertilize oocytes, and also damages DNA (33). Furthermore, spermatozoa are particularly vulnerable to oxidative stress because of their small cytoplasm and limited antioxidant protection.

Sperm chromatin defect could be quantified by acid denaturation by acridine orange and sperm apoptosis by light and electron microscopy. Apoptosis could be determined through morphological criteria (uniform nuclear basophilia with condensation of chromatin or fragmentation of the nucleus into basophilic masses) using microscopy (43) that highlight abnormality of the tail, with hypertrophy and hyperplasia of the flagella, defects of the microtubular doublets in patients with high ROS level and antioxidant deficiency as already described for zinc and NAC.

In 2004 Moustafa et al. (87) have demonstrated that the sperm going to apoptosis, is strongly correlated to ROS level in the ejaculate and this mechanism is more frequent in patients with high level of DFI than in healthy donor patients. Epifluorescent microscopy is a useful tool for studying seminal DNA alterations as well as the electron microscope.

These findings are in line with other studies who found many information such as chromatin, plasma and acrosome membrane structure by electron microscopy. Aydin et al. (88) demonstrated alteration of the acrosome integrity in smoker patients and disruption of the neck region and tail in the freeze/thawed sperm.

There is a growing scientific interest in the mechanisms of containment and self-selection of altered spermatozoa. One of this molecular process still under study concerns cellular autophagy.

For years, the researchers reported the apoptosis like the only mechanisms of planned death cell. In the last studies, autophagy was also described as another process: it seems that it is triggered in response to several insult like heat, radiation, genotoxic substances (exogenous sources) and development of ROS (endogenous sources); this induced a DNA damage that give rise to little cytoplasmic organelles agglomerated in vesicles (called autophagosome) that will be degraded by autolysosome. This programmed cell death seems to be crucial for physiological maturation of spermatogonia and subsequent embryo development avoiding final maturation of stem cells with high level of fragmented DNA (89, 90).

Indeed, unhealthy lifestyle features, including smoking, obesity and poor diet, as well as environmental factors like pesticides and pollution can contribute to develop high oxidation levels. For this reason, a recommended diet requires the supplementation of vegetables and fruits, whole-grain, and fibre-rich products (10).

Furthermore, cross-sectional studies show that a sedentary lifestyle is associated with low semen quality, whereas self-reported increased physical activity appears to be associated with increased motility and total sperm count, also for a reduction of general oxidative stress thanks to sporting activity (91). An unhealthy diet characterized by an excessive intake of saturated fats and a high glycaemic index could directly lead to an enhanced oxidative stress (92–94).

Heavy alcohol consumption is responsible for oxidative stress generation and can induce damage on male reproduction. It can affect male fertility by reducing sperm count, motility and spermiogenesis quality, and inducing morphological abnormalities in sperm (95); ethanol significantly increases the rate of DNA fragmentation in sperm (96) and evidence exists on the role of ethanol as toxin for Leydig cells (97).

One of the main players of the pathological mechanisms at the base of impaired fertility in heavy drinkers is acetaldehyde, which, by interacting with proteins and lipids, triggers ROS generation; the consequent oxidative stress is responsible for an altered spermiogenesis (98).

Ethanol may act not only at the testicular level, but also on the endocrine system, altering the hypothalamic–pituitary–testicular (HPT) axis (99–101).

Nevertheless, the dose-dependent and duration-dependent damages induced by ethanol on male fertility are still controversial. A recent study demonstrated how normal sperm parameters are recovered 3 months after the stop of alcohol intake (102).

In addition, alcohol consumption limited to a period of 4 to 7 weeks may not negatively affect sperm parameters (103, 104).

Regarding antioxidants’ protective roles against ROS-induced damage, their use seems to reduce sperm DNA fragmentation when compared to placebo. Thus, antioxidants could represent an adjuvant treatment in such cases, avoiding further damage caused by ROS (105).

In this context, the improvement of the patient’s antioxidant defences may be important as the excessive generation of ROS in determining the redox balance. In order to address this problem, antioxidant supplementation has been investigated in the treatment of male infertility, but the literature has been invalidated by the lack of data in terms of statistical quality of the studies and sizes of studied populations. However, due to the wide range of antioxidants on the market and the frequent mixing of the different molecules in the same pill, it is difficult to understand the role of each individual component in terms of improving the DFI before and after treatment: collectively, results of antioxidant interventions remain equivocal, and the comparison of the studies is restricted by differences in baseline characteristics, periods of intervention, and methods to evaluate the outcomes.

The most recent Cochrane meta-analysis in 2019 included 5 trials with a variety of antioxidants with a total of 254 subjects, and highlighted that men treated with antioxidants had 5% lower DNA fragmentation (CI) (33). The metanalysis indicates that the current evidence on use of antioxidants in sub-fertile men is inconclusive due to the low quality of the evidence. Nevertheless, despite low-quality evidence, increased live birth rates in case of antioxidant intervention were reported; thus, there is a need for more well-conducted studies in the field of antioxidants (33).

In addition to use *in-vivo*, some authors investigated the effects of antioxidant-supplemented sperm culture media and its power to reduce DNA damage. Some studies have reported that, enriching the freezing medium with various antioxidants (e.g., vitamin E, glutathione, l-cysteine, l-carnitine, various amino acids, SOD, and others) ameliorates sperm parameters (e.g., motility, viability, DNA integrity, acrosomal reaction, and membrane integrity) and reduces the production of ROS during thawing techniques (106).

Other antioxidant therapy like resveratrol or lycopene seems to have a powerful anti-tumoral, antioxidant, anti-inflammatory and anti-apoptotic properties. This molecule displays a wide range of action modulating various processes like immune responses, autophagy and cellular differentiation (25). The biological characteristics of these molecules have made it possible to extend the indication for their use also to patients with seminal pathologies both per os treatment (107) and *in vitro* seminal fluid (108) with statistically significant improvements, albeit to be confirmed in larger and more representative studies.

In recent years, the use of testicular sperm recovery has become more frequent. As already explained, testicular sperm could have lower levels of DNA damage and better DNA integrity than ejaculated sperm. Esteves et al. (72) reported that testicular sperm has three-to-five times fewer fragmentated DNA compared to ejaculated sperm. Afterwards, these data were confirmed by several studies.

The main reason why andrologist surgeons offer the testicular retrieval of sperm to sub-fertile male patients without azoospermia is to increase the chances of live births, in an effort to avoid any possible DNA damage to the sperm during the passage through the reproductive tract.

Better reproductive outcomes were observed in patients whose testicular sperm was used for ICSI, even though the use of antioxidant therapies did not reduce the levels of sperm DNA damage. The birth rate reported in the ICSI group that used testicular sperm was higher, while the relative risk for miscarriage was lower compared to patients who used ejaculated sperm (71).

However, there is currently insufficient evidence to guide a clinical decision. To help clinicians making the right choice, future studies should use a randomized design, avoiding selection bias. It would also be useful to standardize the definition of high or normal DFI by adopting universal criteria for classifying patients with commonly used and accessible tests. Furthermore, it cannot be excluded that the prognostic value of sperm DFI on the outcome of ART techniques is partially biased by the quality of the oocyte and its ability to repair DNA damage (109).

Despite the documented association between male infertility and DFI, this topic remains controversial. In fact, the routine use of sperm DNA fragmentation assays is not recommended by American Society of Reproductive Medicine (ASRM) in the assessment of infertile couples, due to the lack of statistically sufficient evidence supporting the association between DNA fragmentation and II level ART procedures (Practice Committee of the American Society for Reproductive Medicine, 2013). Still, this recommendation may not address couples affected by recurrent pregnancy loss or with history of multiple IVF cycles failure.

Finally, some doubts have been raised about the use of testicular sperm concerning genetic and epigenetic risks on the embryos (69). Nonetheless, retrospective collections of data on infants born from sperm of obstructive and non-obstructive azoospermic men did not show any significant difference in short-term outcomes as well as in the rate of congenital malformations in ICSI progeny from testicular sperm (70).

In summary, to date, many studies have assessed treatments for patients with high DFI. However, the two alternatives are very different, both in terms of psychological acceptance by the patient and in terms of healthcare costs. In fact, if, on one side antioxidants bring no adverse effects, on the other surgical sperm retrieval represents an invasive procedure and, therefore, it is associated with potential complications.

To our knowledge, there are no clinical studies that randomized patients by comparing the results of assisted reproduction techniques after treatment with oral antioxidants vs the use of testicular sperm, that could be helpful to introduce the most valid therapeutic choice.

## Author contributions

MR: Conceptualization, Formal analysis, Funding acquisition, Investigation, Methodology, Project administration, Supervision, Visualization, Writing - original draft, Writing - review & editing; FC: Project administration, Supervision, Visualization, Writing - original draft, Writing - review & editing. DS: Conceptualization, Formal analysis, Funding acquisition, Investigation, Methodology, Project administration, Supervision, Visualization, Writing - original draft, Writing - review & editing. AB: Project administration, Supervision, Visualization, Writing - original draft, Writing - review & editing. EA: Project administration, Supervision, Visualization, Writing - original draft, Writing - review & editing. SC: Project administration, Supervision, Visualization, Writing - original draft, Writing - review & editing. PL-S: Conceptualization, Formal analysis, Funding acquisition, Investigation, Methodology, Project administration, Supervision, Visualization, Writing - original draft, Writing - review & editing. All authors contributed to the article and approved the submitted version.
